# Editorial: Secondary Respiratory Infections in the Context of Acute and Chronic Pulmonary Diseases

**DOI:** 10.3389/fimmu.2019.02764

**Published:** 2019-11-27

**Authors:** François Trottein, John F. Alcorn

**Affiliations:** ^1^Centre d'Infection et d'Immunité de Lille, Inserm U1019, CNRS UMR 8204, University of Lille, CHU Lille, Institut Pasteur de Lille, Lille, France; ^2^Department of Pediatrics, UPMC Children's Hospital of Pittsburgh, Pittsburgh, PA, United States

**Keywords:** respiratory viruses, influenza, COPD, cystic fibrosis, sepsis, pneumonia, innate immune response, immunotherapy

Despite major advances in the identification of key pathophysiological mechanisms and in treatment, respiratory bacterial infections arising from pulmonary insults remain a major clinical issue today. Acute or chronic, sterile or infection-driven, pulmonary inflammation predisposes to secondary respiratory tract infections. Secondary bacterial infections often result in lethal synergy with primary infection in the lung or, in the case of sepsis, throughout the body. Different mechanisms are implicated in this enhanced susceptibility including loss of barrier integrity and impaired host defenses. Today's treatments of secondary bacterial infections are still not effective enough and antibiotic resistance is a major issue. Hence, there is an urgent need for novel therapies. A better understanding of the mechanisms of why secondary bacterial infections arise is of key importance in order to propose novel interventional strategies. In this Research Topic, a series of reviews and original articles provide a timely survey of mechanisms leading to respiratory tract bacterial infections that occur following pulmonary insult including viral (mostly influenza) infections, cystic fibrosis, chronic obstructive pulmonary disease (COPD), and sepsis.

Viral infections predispose patients to secondary bacterial infections, which often have a more severe clinical course. The mechanisms underlying post-viral bacterial infections are complex, and include multifactorial processes mediated by interactions between viruses, bacteria, and the host immune system. Significant advances have been made in recent decades as illustrated by several reviews in this Research Topic. Morgan et al. review the current knowledge about mechanical and immunological mechanisms leading to bacterial super-infection post-viral infections. The authors present the emerging literature describing the role of innate immune cell suppression in secondary bacterial complications. They provide an overview of the principal functions that these cells play in pulmonary immunity, highlighting their unique ability to sense environmental factors and promote protection against respiratory bacterial infections. In particular, the authors discuss mechanisms through which respiratory viruses alter the beneficial cross-talk between airway epithelial cells and macrophages. The role of apoptotic cell clearance (efferocytosis) and reduced responsiveness of pattern recognition receptors (innate imprinting) following viral infection in susceptibility to secondary bacterial infections is highlighted. The authors discuss the importance of extracellular matrix alterations (elevated production following severe acute viral infection) in bacterial attachment, colonization and infection. Finally, they propose different areas for potential new investigation. Paget and Trottein focus on unconventional T lymphocytes (NKT cells, γδ T cells, and MAIT cells) in pulmonary defense against bacterial infection and review mechanisms leading to their dysfunctions in the context of viral-bacterial super-infection. The impact of the antiviral response on unconventional T cell-mediated anti-bacterial host defense is detailed. Finally, they discuss recent advances and future therapeutic opportunities based on the targeting of these cells to prevent bacterial super-infection post-influenza. Hanada et al. discuss the effect of prior viral (influenza) infection on the intestinal and (upper and lower) respiratory tract microbiomes. In this review, the authors summarize the literature on the interactions between host microbial communities in the lung mucosa and host defense. Data generated from mouse models of influenza and from human studies are presented. They also discuss mechanisms through which respiratory viruses disrupt these interactions and contribute to the pathogenesis of secondary bacterial infections. Using examples drawn from current literature, the authors highlight the great complexity of the field and how far we still need to go before proposing microbiome-based therapy. Kiedrowski and Bomberger summarize the current knowledge about the interactions between viruses and bacteria in the cystic fibrosis upper and lower respiratory tract and how super-infections impact the health of individuals with cystic fibrosis. The authors discuss the altered immune and metabolic states in the cystic fibrosis lung due to persistent inflammation and how this may impact bacterial infections. The link between viral exacerbations of cystic fibrosis and bacterial acquisition and outgrowth are covered in detail. Similarly, Wang et al. focus their review on dysregulated inflammation and altered reactive oxygen species production as central causes for acute and chronic bacterial infections in the context of COPD. In the COPD lung, persistent impairment of lung phagocytes results in protease imbalance and tissue damage. The authors go on to focus on interactions between serum amyloid A and formyl peptide receptor 2 (FPR2), which promotes neutrophil chemotaxis and survival. FPR2 signaling in this fashion inhibits the function of resolvin D1, a fatty acid metabolite with anti-inflammatory properties. Potential targeting of resolvin D1 is speculated as a method to limit COPD exacerbations. Finally, two reviews focus on bacterial (nosocomial) super-infection in the context of sepsis which is also characterized by depressed host (pulmonary) defenses. First, Denstaedt et al. recapitulate the dynamics of the septic host response and the balance of inflammatory and anti-inflammatory cellular programs that occur. The authors summarize the epidemiology of nosocomial infections and characteristic immune responses associated with sepsis, as well as immunostimulatory therapies currently under clinical investigation. Sepsis inhibits host defense in distal organs through a variety of pathways including: decreased cytokine production, cytokine receptor antagonism, impaired pattern recognition receptor signaling, suppressor cell activation (myeloid-derived suppressor cells, regulatory T cells), induction of T cell exhaustion, and epigenetic reprogramming. The potential to target these immune deficiencies is discussed in detail. Complementary to this work, Bouras et al. focus their review on dendritic cell paralysis during sepsis and in their contribution to sepsis-induced immunosuppression. They describe the underlining mechanisms involved and propose a set of interventional strategies to overcome this process. Finally, Paolicelli et al. discuss the complexity of the epithelial barrier and introduce the use of lung organoids to better understand interleukin (IL)-17 signaling and host defense against bacterial infections. The relative strengths and weaknesses of air-liquid interface epithelial cell cultures, lung on a chip technology, and lung epithelial organoids are discussed. The use of these approaches will provide critical insight into cytokine, epithelial cross-talk in host defense against extracellular pathogens.

In original research articles, Shepardson et al. and Gopal et al. focus on type I interferons (IFN), a family of cytokines that regulate both anti-influenza immunity and host susceptibility to subsequent bacterial super-infections. The type 1 IFN/signal transducer and activator of transcription 1 (STAT1) axis was previously shown to inhibit type 17 immune response resulting in exacerbation of bacterial burden and mortality during influenza and bacterial (pneumococcal and staphylococcal) infections. Gopal et al. demonstrated that STAT2, which is required for type I and type III IFN signaling and virus clearance, participates in bacterial super-infection. In this setting, STAT2 regulates macrophage phenotype and suppresses bactericidal activity. Deletion of STAT2 during influenza results in increased numbers of dual M1/M2 marker expressing macrophages in the lung that demonstrate improved bacterial clearance. Despite impaired anti-viral host defense in STAT2-deficient mice, these mice are protected from super-infection induced mortality. Shepardson et al. investigate the role of type I IFN receptor (IFNAR2). While the impact of IFNAR1 signaling in influenza and super-infection contexts has been reported, little is known about the specific role or IFNAR2. The authors show that IFNAR2-deficient mice have significantly impaired anti-viral host defense and have increased morbidity and mortality following influenza challenge. In super-infection, IFNAR2 appears to play a divergent role compared to IFNAR1 depending upon timing of bacterial challenge. At day 3 post-influenza infection, IFNAR2-deficient mice do not display altered susceptibility like IFNAR1-deficient mice. However, when challenged with bacteria at day 7 post-influenza, IFNAR2-deficient mice are protected similar to IFNAR1-deficient mice. These two studies illuminate as of yet unknown details regarding interferon signaling and susceptibility to secondary infections. Using a novel influenza A virus/*Klebsiella oxytoca* super-infection model, Lee et al. investigate host resistance and host tolerance, two processes essential for host survival during infection. In this system, the authors show that combined dysfunctional tolerance and resistance mechanisms cause worsened outcomes for the host. The authors identify *K. oxytoca* as a component of the human microbiome and cause of secondary infection post-influenza. Several unique features of this model are described including, delayed bacterial clearance, increased M1 macrophage activation, and, despite a small impact on lung leak, increased mortality vs. influenza infection alone. Finally, Jubrail et al. investigate the response of macrophages to human rhinovirus, a virus frequently isolated from COPD patients during exacerbations. Little is known about mechanisms of secondary bacterial infections with regard to non-influenza viruses. In this study, prior viral exposure blunts the macrophage responses and cytokine production to *Haemophilus influenzae*. This paralyzed macrophage phenotype was not observed in response to bacterial stimuli alone and likely indicates commonalities between influenza and other respiratory virus induced super-infections. Additional work is necessary to determine if the immune pathways of susceptibility implicated in influenza super-infection can be generally applied to the myriad of common respiratory viruses.

To conclude, recent advances in the field have sparked interest in the role of pulmonary injury and inflammation in secondary bacterial pneumonia. By expanding this knowledge base and understanding, researchers and clinicians hope to pave the way toward devising strategies to positively modulate lung immune responses within diverse clinical scenarios to combat against opportunistic bacteria. It is clear that from a mechanistic and public health point of view, such studies will be important because of the constant aging of the population, antibiotic resistance, and limits of vaccine efficacy. Immunomodulatory therapy has become increasingly important in the treatment of cancer and auto-immune diseases. Its potential in the context of acute pulmonary illness is only now emerging. The majority of the mechanistic information that we now know regarding secondary bacterial infections has been demonstrated in the mouse model. While complex polymicrobial infections have been studied in chronic lung disease in humans, very little is known in the acute viral infection context. Translational studies are needed to determine the conservation of susceptibility mechanisms to bacterial super-infections between mice and humans in order to advance therapeutic options in support of improved clinical care.

## Author Contributions

All authors listed have made a substantial, direct and intellectual contribution to the work, and approved it for publication.

### Conflict of Interest

The authors declare that the research was conducted in the absence of any commercial or financial relationships that could be construed as a potential conflict of interest.

